# Zinc knuckle of TAF1 is a DNA binding module critical for TFIID promoter occupancy

**DOI:** 10.1038/s41598-018-22879-5

**Published:** 2018-03-15

**Authors:** Elizabeth C. Curran, Hui Wang, Thomas R. Hinds, Ning Zheng, Edith H. Wang

**Affiliations:** 1 Department of Pharmacology, University of Washington, Seattle, WA 98195 USA; 2 Howard Hughes Medical Institute, University of Washington, Box 357280, Seattle, WA 98195 USA

## Abstract

The general transcription factor IID (TFIID) is the first component of the preinitiation complex (PIC) to bind the core promoter of RNA polymerase II transcribed genes. Despite its critical role in protein-encoded gene expression, how TFIID engages promoter DNA remains elusive. We have previously revealed a winged-helix DNA-binding domain in the N-terminal region of the largest TFIID subunit, TAF1. Here, we report the identification of a second DNA-binding module in the C-terminal half of human TAF1, which is encoded by a previously uncharacterized conserved zinc knuckle domain. We show that the TAF1 zinc knuckle aids in the recruit of TFIID to endogenous promoters vital for cellular proliferation. Mutation of the TAF1 zinc knuckle with defects in DNA binding compromises promoter occupancy of TFIID, which leads to a decrease in transcription and cell viability. Together, our studies provide a foundation to understand how TAF1 plays a central role in TFIID promoter binding and regulation of transcription initiation.

## Introduction

The formation of the preinitiation complex (PIC) is a step-wise process required for eukaryotic transcription of protein-encoded genes^1,2^. The highly-orchestrated assembly of the general transcription factors (GTFs) TFIIA, B, D, E, F, and H is responsible for properly positioning RNA polymerase II (RNAPII) at core promoters and initiating transcription. TFIID nucleates PIC formation by directly binding to promoter sequences. TFIIA further stabilizes this interaction, while TFIIB association is firmly established by TFIIF/Pol II recruitment, and TFIIE/TFIIH are essential for promoter clearance^3,4^.

TFIID is a multi-subunit complex comprised of the TATA binding protein (TBP) and 14 TBP associated factors (TAFs) in humans^5^. A small subset of these factors recognizes specific core promoter elements (CPEs) to orient the PIC at the transcription start site^6–8^. TFIID’s ability to selectivity recognize promoter sequences was initially thought to be largely dictated by TBP binding to the TATA box^9^. The primary evidence for this model centers on TBP’s ability to support a low level of transcription in the absence of TAFs, whose roles include responding to transcriptional activators^10,11^. Growing evidence supports the idea that TBP alone does not identify gene promoters, especially in higher organisms^5,11,12^. The majority of human protein-coding genes lack a TATA box sequence, yet TFIID is still able to engage the initiator and direct transcription from TATA-less promoters^13^. Therefore, other PIC components must be involved in dictating DNA specificity. A recent study demonstrated the TAF components of TFIID are vital for sequence selectivity while TBP alone showed no sequence preference^14^. This expands upon previous DNA footprinting analyses that illustrated an extended region of protection by TFIID compared to TBP, which was interpreted as TAFs binding near the transcriptional start site and downstream promoter sequences^6,15^.

The specific combination of CPEs varies from gene to gene, and their unique arrangement may help to ensure genes are expressed at specific times in response to cellular needs. Most of these elements are recognized by TAFs to enhance the interaction between TFIID and promoter DNA^2^. The Initiator (Inr) is positioned at the transcription start site of over 40% of human protein-encoding genes and is enriched at TATA-less promoters^16,17^. Motif ten element (MTE), downstream promoter element (DPE), and downstream core element (DCE) are located downstream of the transcription start site and thought to contribute to promoter recognition in the absence of a TATA box^2,18,19^. TAF1, the largest subunit of TFIID, has been shown to associate with the DCE, a discontinuous sequence found in viral promoters as well as the human beta globin gene^19^. TAF1 also interacts with the Inr when in complex with TAF2^6^. While there is a wealth of knowledge about promoter sequences, little is known about TAF/DNA binding interfaces and their mode of interaction. Characterizing these protein-DNA interactions will advance our understanding of how the entire TFIID complex mediates transcriptional initiation.

Our previous work describing a winged helix (WH) located in the central DUF3591 domain provided the first look at TAF1’s promoter recognition capabilities^20^. When our structure of TAF1 DUF domain bound to TAF7 was modeled into the cryo-EM structure of promoter bound TFIID, it confirmed the WH can contact downstream promoter sequences^21^. The cryo-EM structure further revealed a small secondary density of protein contacting the Inr, which the authors attribute to TAF1. The advancements in cryo-EM have given us a general idea of the overall shape of TFIID and insights into the topological rearrangements that ensue upon promoter binding^21,22^. Yet, further refinements are necessary to definitively resolve all the crucial interfaces between the TAF subunits of TFIID and core promoter sequences during the dynamic structural reorganization evoked by promoter engagement.

The idea that TAF1 contains multiple DNA binding domains is supported by several pieces of evidence. In addition to structural data, TAF1 has been shown to bind two entirely distinct and separate promoter elements (i.e. Inr and DCE) implying multiple binding modules^6,19^. The DCE is a cluster of three non-contiguous sequences. Each was shown to be important for transcription and can crosslink to TAF1; the current model suggests TAF1 directly binds the entire DCE, a region too large to be bound by the WH alone. Moreover, yeast TAF1 has been shown to contain a promoter-binding region near its C-terminus outside the WH^23^. This region corresponds to sequence adjacent to the double bromodomain in the human homolog, and yeast TAF1 lacking this region was unable to bind promoter DNA. Multiple DNA binding domains in TAF1 may convey plasticity to allow recognition of diverse promoter sequences and permit structural rearrangements during promoter engagement.

Hidden between the double bromodomain and the DUF domain of TAF1, we discovered a strictly conserved CCHC zinc knuckle (ZnK) that plays a vital role in transcriptional regulation. By examining human TAF1 ZnK function under both native and *in vitro* conditions, we identified a second previously uncharacterized DNA binding module in the largest subunit of TFIID. Our study shows that TAF1 ZnK is important for cellular viability and helps to direct TFIID to endogenous core promoters through its DNA binding abilities. This work establishes a basis for dissecting the complexity of TFIID promoter recognition.

## Results

### TAF1 contains an evolutionarily conserved zinc knuckle motif

**Figure 1:**
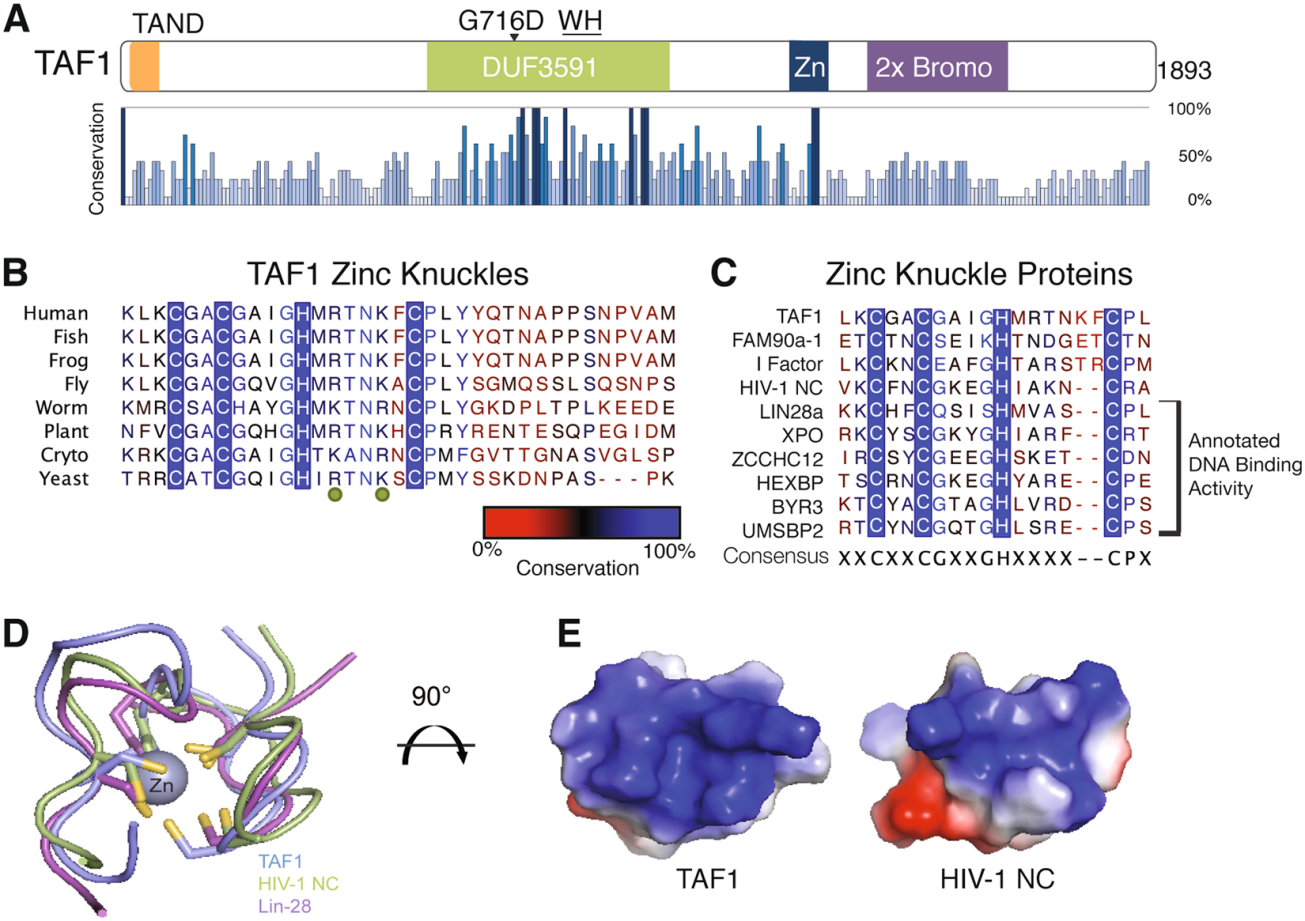
TAF1 contains an evolutionarily conserved zinc knuckle. (**A**) Linear schematic of full length human TAF1 with percent conservation in eukaryotes shown. (**B**) Annotated TAF1 zinc knuckle conservation alignment spanning vertebrates, insects, nematodes, plants, and fungi. Zinc knuckle cysteines and histidine are boxed in blue. Conserved positive residues indicated by the green dots. (**C**) Sequence alignment of zinc knuckle containing proteins from eukaryotes and viruses. (**D**) Alignment of TAF1 ZnK model (blue) from I-TASSER prediction analysis with known structure of ZnK of HIV-1 nucleocapsid protein (green) and lin28 (purple); (**E**) Electrostatic surface map of HIV-1 nucleocapsid protein nucleic acid binding surface (right) and corresponding region of TAF1 ZnK (left).

TAF1 is an essential protein found in all eukaryotic organisms. The preservation of TAF1 across these kingdoms signifies its biological importance. By using sequence information and mapping regions of high conservation, we identified portions of the protein that were selectively maintained throughout evolution. A sequence alignment performed on full-length TAF1 using eight species that span the eukaryotic kingdom revealed two domains characterized by strictly conserved residues, which are absent in other regions of the protein (Fig. 1A). The DUF3591 domain is the largest region of conservation. Notably, in the *ts13* mutant cell line, this domain harbors a temperature-sensitive point mutation (G716D) that causes cells to cell cycle arrest in late G1 and eventually undergo apoptosis^24–26^. We previously characterized the structural features of this domain with its binding partner TAF7 and discovered a winged-helix (WH)^20^. The second region of strict conservation sits between DUF3591 and the double bromodomain (2 × Bromo). Zooming in on the amino acid sequence within this region reveals an invariable Cx_2_Cx_4_Hx_6_C motif (Fig. 1B). Bioinformatics predicts this motif as a putative zinc knuckle (ZnK), a common bimolecular interacting domain.

The CCHC ZnK is a widely occurring domain commonly found in nucleic acid binding proteins^27–29^. A large fraction of CCHC-domain containing proteins, which can be aligned with TAF1 ZnK, are involved in DNA binding, hinting that TAF1 ZnK also may interact with DNA (Fig. 1C). In addition to the zinc coordinating residues, several other amino acids are common to the majority of CCHC motifs: glycine following the second cysteine, glycine preceding the histidine, and proline after the third cysteine. The consistent location of these residues suggests that they may be important for proper domain folding. The spacing of the first two cysteines and histidine is strictly conserved; however, the spacing between the histidine and third cysteine can vary. Interestingly, TAF1 shares the same spacing of two proteins: I-factor from *Drosophila* and FAM90a in humans^30–32^. I factor is a LINE-like transposable element and contains a ZnK in ORF1, which has been shown to bind DNA^31^. FAM90 is a protein family found in primates, thought to have arisen during multiple duplication and rearrangement events^32^. The ZnK in FAM90 has been proposed to function as a DNA binding domain. The commonality of DNA binding to this similarly spaced group of zinc knuckle proteins further suggests that TAF1 ZnK may interact with DNA.

A structural model of TAF1 ZnK was generated using I-TASSER^33^ and shows a compact fold with the C-C-H-C side chains pointing towards the center, allowing for coordination of a zinc ion (Fig. 1D). Our analysis estimated the probability of ligand binding for the C-C-H-C to be above 90% based on the COACH binding prediction. A structural homology search identified HIV-1 nucleocapsid protein and lin-28 as top hits. Both proteins are known to interact with nucleic acid, and solution structures of these proteins bound to RNA have been solved^28,34,35^. An alignment between these structures reveals substantial similarities in the zinc coordinating residues as well as overlapping charged residues (Fig. 1D). Charged surface analysis on ZnK of TAF1 identified one side of the ZnK fold that is enriched with positively charged residues with positive electrostatic potential (Fig. 1E). Positive electrostatic patches are a highly predictive hallmark of DNA binding proteins. Furthermore, this charged surface displays considerable similarity to the HIV-1 nucleic acid binding surface and contains two strictly conserved positively charged amino acids that may be critical for electrostatic interactions (Fig. 1E). These outward facing positively charged residues produce an interface to presumably interact with negatively charged nucleic acids. Taken together, these features signify TAF1 ZnK has the potential to function as a DNA binding domain and thus contribute to TFIID promoter recognition.

### TAF1 zinc knuckle motif is essential for cell viability

**Figure 2:**
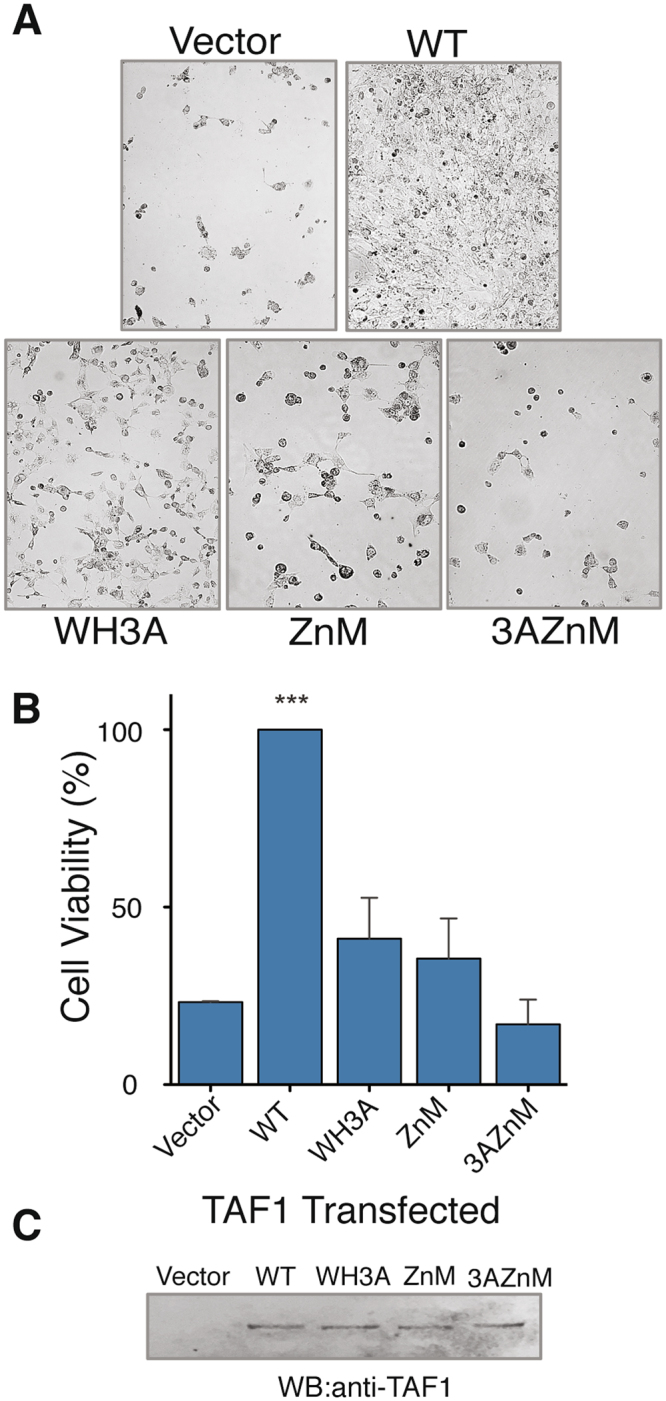
TAF1 zinc knuckle is required for cell viability. (**A**) Phase contrast images of ts13 cells transfected with pCS2 + (vector), wild type TAF1 (WT), winged-helix mutant (WH3A), zinc knuckle mutant containing two cysteine to alanine mutations (ZnM), and double mutant (3AZnM) at 39.5 °C for 72 hrs. (**B**) Quantitation of viable DAPI stained cells (n = 3). Error bars represent standard deviation. Two-tailed analysis compared to vector with a 95% confidence, ***p < 0.0001 (Unpaired t-test). (**C**) Western blot of ts13 lysates expressing HA-tagged TAF1 proteins. Proteins were immunoprecipitated with TAF1 specific double bromodomain antibody and immunoblotted for exogenously expressed TAF1 using anti-HA antibody. The full-length blot is presented in Supplemental Figure S4.

Given the strict conservation of the TAF1 ZnK domain, we asked whether it plays a physiological function in cells. We used the *ts13* cell viability assay to determine if the ZnK domain is required to complement the *ts13* temperature-sensitive G1 growth defect. *ts13* cells contain a conditional mutation in TAF1 and proliferate at the permissive temperature of 33.5 °C^24,25^. When shifted to 39.5 °C, the cells arrest in late G1 and undergo apoptosis unless a functional copy of TAF1 is exogenously expressed^25,36,37^. Cells transfected with wild-type TAF1 continue through the cell cycle and proliferate at the elevated temperature. Conversely, non-functional TAF1 results in a decrease in the number of viable cells. We chose to mutate the first two cysteines in the ZnK domain of TAF1 to ensure the motif was fully compromised. Changing the cysteines to alanines resulted in a ~60% reduction in cell viability, indicating a structurally intact ZnK is vital for full physiological function (Fig. 2A,B). The level of cell survival was comparable to point mutations in the previously characterized TAF1 winged helix (Fig. 2B). Moreover, combining the ZnK and WH mutations (3AZnM) tended to further reduce cell viability compared to the single mutants but the decrease was not statistically significant. This finding can be taken to suggest that both domains must be fully functional for proper TAF1 activity. Importantly, all TAF1 constructs were expressed at equivalent levels according to western blot analysis, indicating that the mutations do not cause protein instability (Fig. 2C).

### Zinc knuckle and winged helix of TAF1 are necessary for cyclin gene transcription

**Figure 3:**
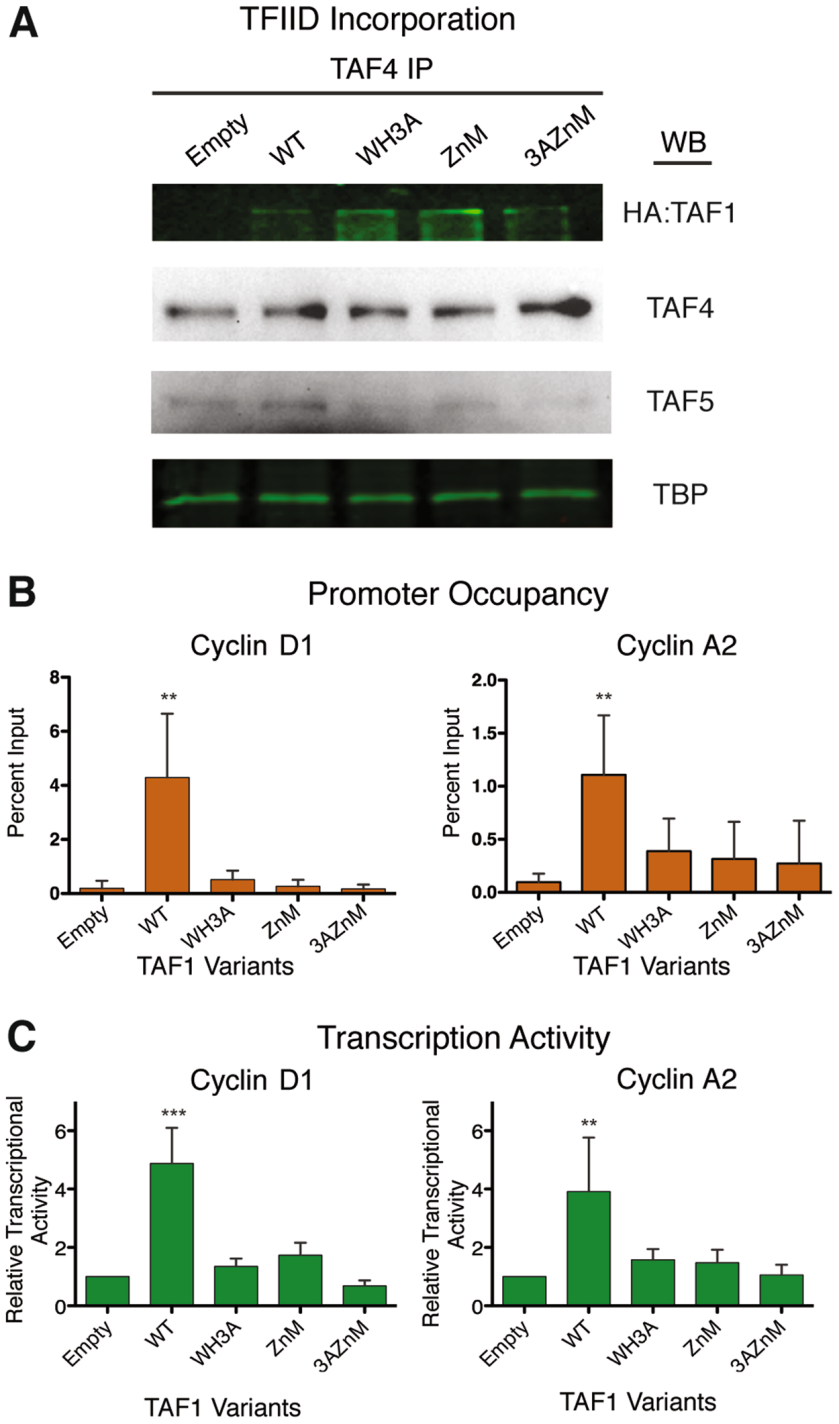
TAF1 zinc knuckle and winged helix are imperative for effective cyclin promoter activity. (**A**) Incorporation of TAF1 proteins into TFIID. TFIID complexes were isolated by immunoprecipitation and incorporated HA-TAF1 variants detected by anti-HA immunoblotting. Additional TFIID subunits immunoprecipitated were detected by immunoblotting. The full-length blots are presented in Supplemental Figure S5. Quantitation of normalized HA-TAF1 and TAF protein levels are provided in Supplemental Table 2. (**B**) Chromatin immunoprecipitation of HA-TAF1 variants expressed in HEK293 cells followed by qPCR for cyclin D1 and cyclin A2 promoters (n = 4). (**C**) Luciferase assay of cyclin D1 and cyclin A2 promoter driven reporter constructs co-transfected with TAF1 variants. Luciferase activity was normalized for total protein and expressed relative to reporter activity without exogenous TAF1 (Empty), given a value of 1.0 (n = 3). All error bars represent standard deviation. Two-tailed analysis compared to WT with a 95% confidence, **p < 0.01, ***p < 0.0001 (Unpaired t-test).

TAF1 is known to be important for transcription of the cell cycle genes cyclin A, D1 and E^36–38^. The failure of ZnM and WH3A to complement the growth defect in *ts13* cells signifies that these mutations possibly compromise a function of TAF1 important for cyclin gene expression. We determined that this functional defect is not due to an inability to incorporate into TFIID, but rather a deficiency in promoter recognition. We verified the TAF1 mutants can successfully integrate into the TFIID complex by expressing each TAF1 variant as an N-terminal HA-fusion protein (HA-TAF1), using an antibody against the TAF4 subunit to immunoprecipitate the entire TFIID complex, and immunoblotting for exogenously expressed TAF1 variants using an anti-HA antibody. The presence of other TFIID subunits was examined by western blotting (Fig. 3A) and silver staining (Supplemental Figure S5). We observed that all TAF1 variants incorporated into TFIID at relatively equal levels compared to the wild-type protein (Fig. 3A). By contrast, the TAF1 mutants did not associate with core promoters of cell cycle genes in chromatin immunoprecipitation experiments (Fig. 3B). For these studies, different HA-TAF1 variants were expressed in HEK293 cells and TAF1-bound promoter fragments were recovered and quantified by qPCR. Cyclin D1 and cyclin A2 were chosen as target promoters because the proper expression of these genes is required for cell cycle progression; their promoter function also has been shown to be dependent on TAF1 activity^37–39^. The specific promoter regions for qPCR amplification were selected based on ENCODE ChIP data for TAF1. Additionally, TAF1 promoter binding is considered synonymous with canonical TFIID binding, because TAF1 is unique to TFIID whereas other TAFs and TBP are found in additional auxiliary transcription regulatory complexes^2,40^. We detected that wild type TAF1 effectively bound to cyclin D1 and A2 promoter regions, while neither the ZnM nor WH3A mutant selectively enriched for these promoter fragments (Fig. 3B). Promoter association of the double mutant (3AZnM) was not significantly lower than either single domain mutant. The overall conclusion is that the WH3A and ZnM mutants consistently bind less efficiently to cyclin genes and that both domains can contribute equally to TFIID promoter recognition.

Next, we determined the impact loss of promoter recognition had on transcription levels from the cyclin D1 and A2 promoters. By leveraging a luciferase reporter assay, a conventional method for studying gene expression, we asked whether exogenously expressed TAF1 variants affected cyclin promoter function. The luciferase gene was placed under the control of the human cyclin D1 or cyclin A2 promoter. TAF1 variants were co-transfected with each luciferase reporter construct, and the amount of luciferase activity, a measure of promoter activity, was assessed approximately 36 hours post-transfection. Reporter activity in the absence of exogenously expressed TAF1 was quantified to assess the endogenous transcriptional activity of the cyclin promoters. This signal was used to normalize across experiments and served as a control ensuring transcription was not negatively impacted by expressing a single TFIID subunit. A nominal increase in TAF1 should enhance TFIID formation because under normal conditions TAF1 is in limited supply compared to other TFIID components^41^. Indeed, the expression of wild type TAF1 significantly increased the level of luciferase activity from both promoters compared to basal levels (Fig. 3C). In contrast, TAF1 with mutations to either the ZnK or WH failed to significantly stimulate the activity of the cyclin promoters above background levels. Similarly, the double mutant had no effect on cyclin promoter activity (Fig. 3C). Hence, there was no statistical difference between all TAF1 mutants and background levels. We also examined the effects of WT and mutant TAF1 expression on transcription from the endogenous cyclin D1 promoter in ts13 hamster cells, which were used for the cell complementation assays. We observed reduced stimulation of cyclin D1 transcription by the TAF1 domain mutants compared to WT TAF1 (Supplemental Figure S1). Collectively, these data suggest ZnK and WH are critical to recruit TFIID to core promoters and transcriptional initiation.

### TAF1 Zinc Knuckle Binds DNA

**Figure 4:**
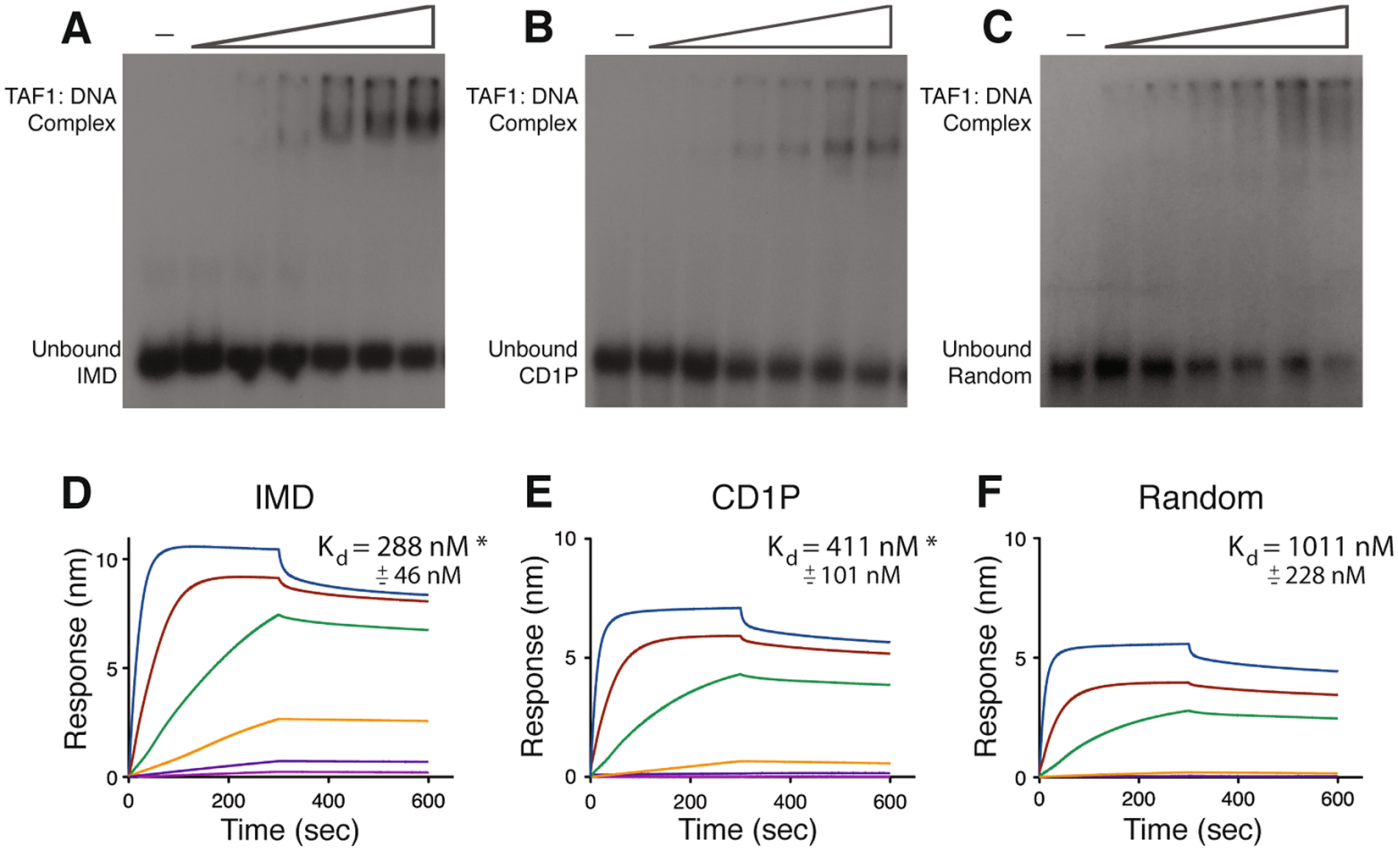
TAF1 zinc knuckle binds DNA. (**A,B,C**) EMSA of TAF1 ZnA (aa 1234–1375) and three radiolabeled DNA fragments: IMD of super core promoter (position −6 to +38), cyclin D1 promoter (position −22 to +29, CD1P), and Random DNA sequence. (**D,E,F**) Bio-layer interferometry binding curves using biotinylated double-stranded DNA fragments described in above and the following ZnA protein concentrations: 3 μM, 1 μM, 333 nM, 111 nM, 37 nM, 12 nM. Raw data was plotted with GraphPAD Prism. K_d_ was calculated from plotting steady-state binding levels against protein concentration.

Structure homology modeling predicts that TAF1 ZnK potentially interacts with nucleic acids. We performed electrophoretic mobility shift assays (EMSA) to determine if the ZnK domain binds DNA. The protein fragment used in this assay contained the annotated ZnK domain and flanking regions (ZnA, aa 1234–1375) so as to not exclude any potential interacting residues. Increasing concentrations of ZnA protein were incubated with three different ^32^P-labeled double-stranded DNA fragments then subjected to native polyacrylamide gel electrophoresis. The fragments represent an optimized promoter containing an Inr, MTE and DPE (IMD); an endogenous promoter, cyclin D1 (CD1P); and a random DNA sequence (Random). We observed that TAF1 ZnA bound to all three DNA fragments (Fig. 4A–C). We followed up this analysis by using an orthogonal technique, bio-layer interferometry (BLI), for a more quantitative measurement of binding affinity. Biotinylated double-stranded IMD, CD1P and Random oligonucleotides were loaded on streptavidin probes and incubated with different concentrations of ZnA protein. Association and dissociation kinetics were monitored in real-time over two consecutive 5-minute periods, respectively (Fig. 4D–F). Dissociation of the protein from the probe indicated that the proteins were not irreversibly aggregating on DNA. Because of the slow off rate observed, binding affinities were calculated based on steady state levels for each protein concentration and involved plotting steady state binding levels against protein concentration for each DNA. ZnA binds significantly better to the optimized promoter (IMD) and CD1P DNA (288 nM ± 46 nM, and 411 nM ± 101 nM, respectively) over random DNA (1011 nM ± 228 nM). This data correlates with the promoter strength of each fragment in luciferase reporter assays, where IMD exhibits the highest activity followed by the CD1 promoter (Supplemental Figure S2). Interestingly, the null construct lacking a promoter sequence is still able to support low levels of transcription indicating general transcription factors are able to bind and initiate transcription from random sequences. The lack of strong specificity may in fact be a necessary feature of TAF1 given the sequence diversity within Pol II promoters. Overall, these studies provide a possible mechanism for how compromising the TAF1 ZnK leads to diminished transcription.

### Zinc Knuckle is critical for DNA binding

**Figure 5:**
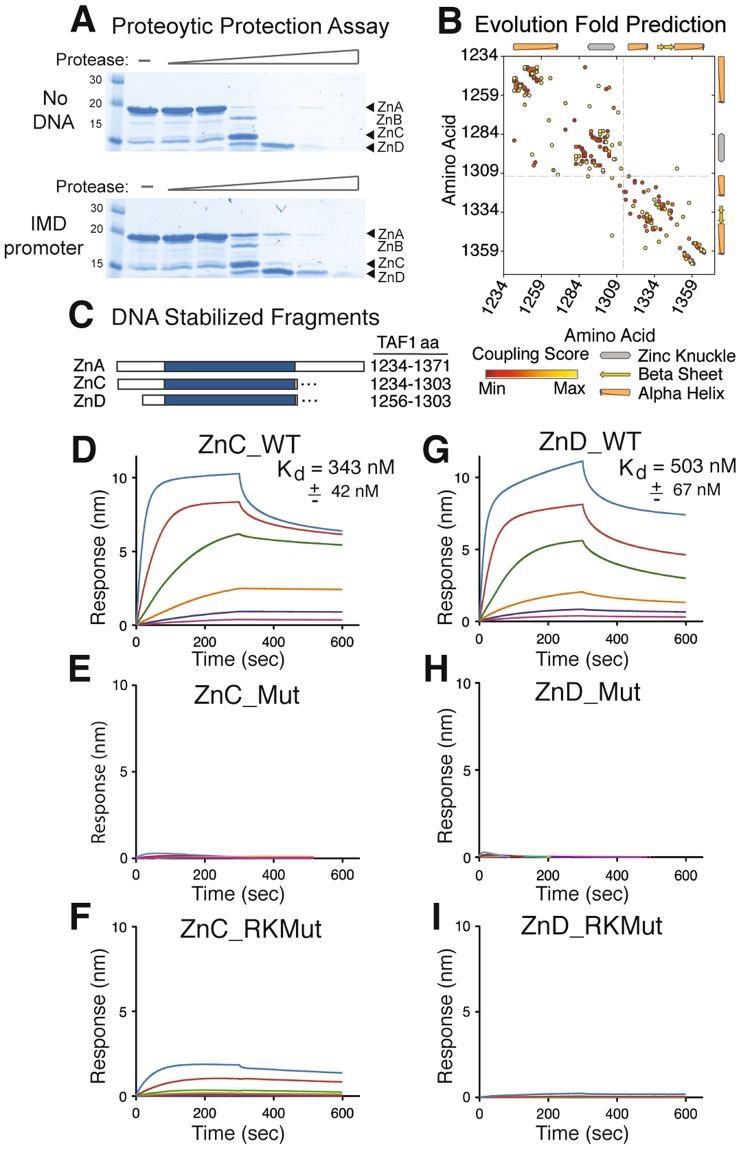
Core Module and Key Residues in TAF1 Zinc Knuckle DNA Binding Domain. (**A**) TAF1 (aa 1234–1375) was incubated without (upper) and with (lower) IMD promoter DNA followed by digestion with increasing concentrations of protease. Digestion products were resolved by SDS-PAGE and detected by coomassie blue staining. Arrowheads indicate fragments stabilized by DNA. Quantification of protein fragments are provided in Supplemental Figure S3. (**B**) EVfold map analysis of ZnA. Numbers represent amino acid residues of full-length TAF1. (**C**) Diagram of DNA stabilized regions of TAF1 with blue box indicating CCHC ZnK. DNA binding curves for (**D**) ZnC wild type, (**E**) ZnC cysteine mutant (C1285A and C1288A), (**F**) ZnC charge mutant (R1295A and K1298A), (**G**) ZnD wild type, (**H**) ZnD cysteine mutant, and (**I**) ZnD charge mutant. Raw data was plotted with GraphPAD Prism. K_d_ was calculated by plotting steady-state binding levels against protein concentration and determining the concentration needed for half maximal binding.

To delineate the minimal ZnK DNA binding domain, TAF1 ZnA protein was incubated with IMD DNA fragment, and the mixture subsequently exposed to increasing concentrations of subtilisin protease. Digestion products were separated by SDS-PAGE, and the pattern of protein fragments compared to the apo-ZnA digestion pattern, generated in the absence of IMD incubation. Protein regions bound to DNA were protected from proteolytic cleavage and led to three stabilized species (Fig. 5A). As anticipated, at the higher concentrations of protease, ZnA is fully degraded as DNA-binding is a dynamic process and the DNA is unable to permanently protect the protein from enzymatic degradation. Quantification of the relative intensity of the different proteolytic products (Supplemental Figure S3) revealed that the full-length construct (ZnA) was considerably stabilized along with two smaller fragments (ZnC and ZnD), which were subsequently N-terminally sequenced. Surprisingly, fragment ZnC began at the same amino acid as the intact ZnA protein (aa 1234). The conversion of ZnA into ZnC, therefore, must be due to cleavage of the C-terminus. The N-terminus of the ZnD fragment was mapped to amino acid 1256, indicating that N-terminal cleavage of ZnC results in ZnD production. Interestingly, ZnC was the least stabilized by the IMD promoter, suggesting that DNA binding more effectively prevents cleavage of the C-terminus than the N-terminus of the ZnA fragment.

To further define the TAF1 ZnK domain, we analyzed ZnA sequence with EVfold, a program that mines evolutionary information to detect connections between residues in a protein and predicts a three-dimensional shape based on the co-conservation of amino acids^42^. Strong connections are given high coupling scores and indicate the residues have a high probability of existing in the same three-dimensional space. Plotting the connections illustrates the distance between co-evolved residues and identifies modular domains within proteins. EVfold analysis revealed the ZnA construct contains two smaller subdomains: the N-terminus from aa 1234–1313, which contains the ZnK, and a C-terminal domain spanning aa 1313–1375 (Fig. 5B). It is very striking that the defining boundary between these two subdomains corresponds to the end of the yeast TAF1 homolog. An alignment of fungal TAF1 proteins reveals that sequence conservation ends three amino acids after the last zinc knuckle cysteine residue. Taken together, we mapped the potential minimal ZnK binding domain to be aa 1256–1303 (ZnD) (Fig. 5C). We then generated two constructs, ZnC (aa 1234–1303) and ZnD, and purified these protein fragments, which were further analyzed by BLI using IMD loaded probes (Fig. 5D,G). The K_d_ values for ZnC (343 nM ± 42 nM) and ZnD (503 nM ± 67 nm) were similar to that obtained for ZnA, indicating that ZnD represents the core domain of DNA binding. As expected, mutations to the strictly conserved cysteines completely abolished binding (Fig. 5E,H). Due to the lack of appreciable signal above background, this binding data could only be used for qualitative assessment. Next, we sought to determine the residues critical for DNA binding. Guided by our predicted structural model, we mutated two positively charged residues between the histidine and third cysteine, which are located on the ZnK predicted DNA binding surface and are conserved across species (see Fig. 1B,E). Consistent with a critical role in binding DNA, mutation of these two positively charged residues either substantially compromised or completely abrogated the DNA binding activity of ZnC and ZnD, respectively (Fig. 5F,I). The residual DNA binding activity of the ZnC_RK mutant is most likely attributable to several additional positively charged residues at the N-terminal region of the ZnC fragment. Together, these experiments demonstrate the capacity of TAF1 ZnK to bind to DNA and map two charged residues essential for this activity. The identification of an additional DNA binding module in human TAF1 implies TFIID employs a multi-level approach to engage DNA, and with this knowledge we will continue to progress our understanding of promoter recognition and transcriptional initiation.

## Discussion

CCHC zinc knuckles are critical physiological domains and are found across the biological spectrum. The majority of ZnKs are involved in nucleotide processing including chaperoning, splicing, transcriptional activation and termination^27–29^. The ZnK found in TAF1 has a unique spacing shared by only a small number of annotated ZnK proteins, I factor and FAM90a, both of which have been annotated as interacting with DNA^31,32^. Given the propensity for zinc knuckles to bind DNA and TFIID’s role in transcription, we set out to determine if TAF1 ZnK interacts with promoter DNA. We found that TAF1 ZnK can bind to DNA with an affinity similar to other zinc knuckles^43,44^ and identified the residues important for this function. The sequence specificity of the ZnK, while significant, is not absolute. This mirrors ChIP-seq studies that fail to identify any sequences enriched by endogenous TAF1. This data strengthens our findings that show a marginal yet significant preference for promoter DNA over a random sequence. TAF1 ZnK is still able to bind random DNA with considerable affinity, 1 μM, suggesting ZnK may bind with high affinity at some promoters and low affinity at others. This also could be argued as a caveat of using an *in vitro* system to understand the complexities of physiological conditions. Follow-up experiments such as ChIP seq with mutant forms of TAF1 could help resolve this proviso and identify any sequences that preferentially bind an intact ZnK domain over WHD. Likewise, structural studies would definitely establish the mechanism of binding to DNA and can be used to clearly visualize if the protein/DNA contacts could allow for flexibility in sequence recognition.

We further show that TAF1 ZnK is important for cellular viability. Mutations in the ZnK lead to a reduction in TFIID association with cyclin D1 and A2 promoter sequences, as shown by a decrease in TAF1 ZnM promoter binding by ChIP and a lack of transcriptional stimulation in luciferase reporter activity assays when compared to wild type TAF1. The discovery of a second DNA binding module in human TAF1 is a notable step forward for our understanding of transcriptional initiation. TAF1 DNA binding activity is a vital yet under studied aspect of transcription. Further elucidation of TAF1’s role in core promoter recognition will have a profound impact on our perception of transcriptional regulation.

**Figure 6:**
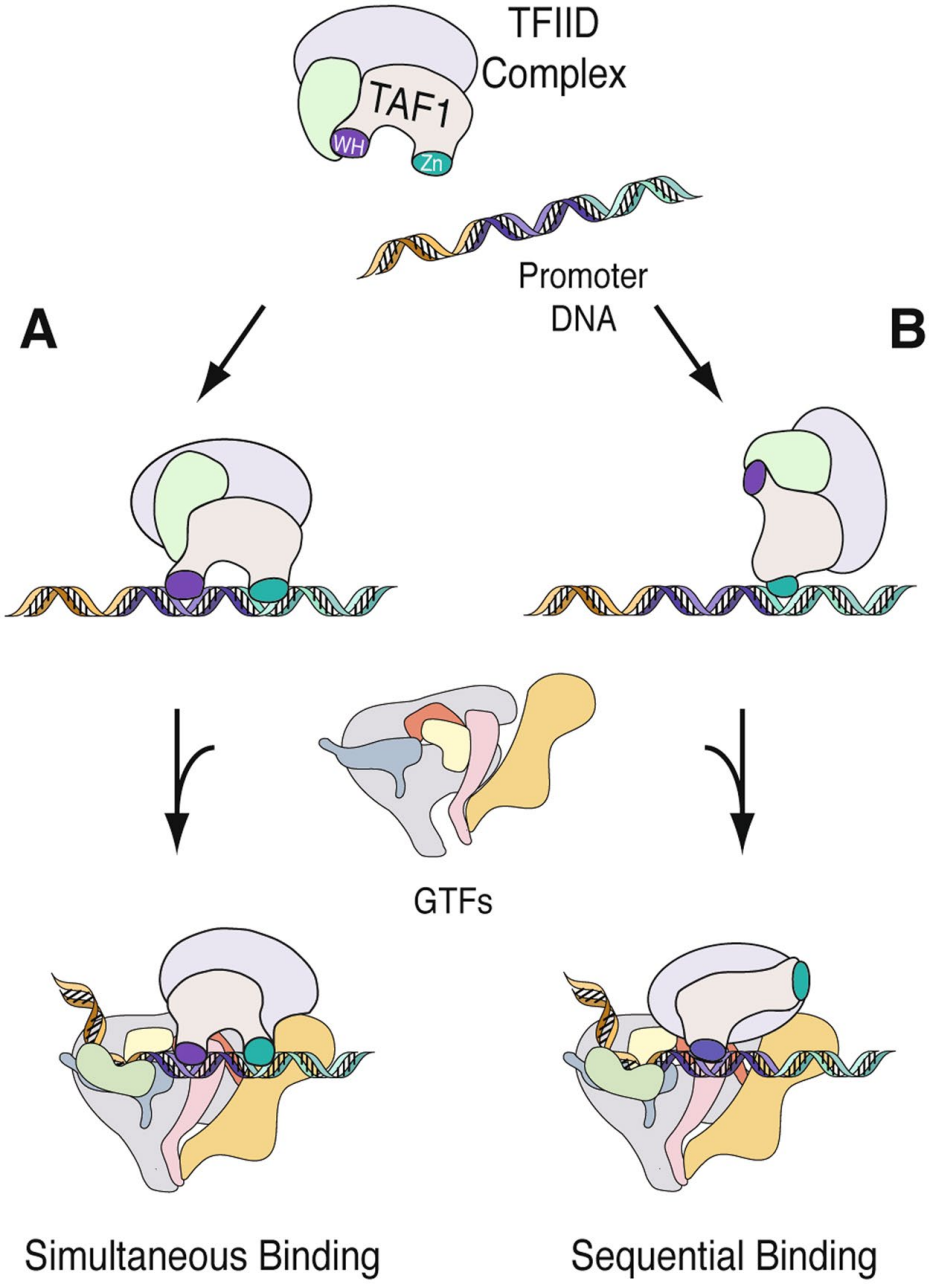
Model of TAF1’s Role in Promoter Recognition. (**A**) ZnK and WH Bind Simultaneously. (**B**) Sequential Binding of ZnK and WH.

TFIID promoter recognition is an essential step in transcriptional regulation, yet we are only beginning to untangle the complexities of its mechanism. Our data identified a new conserved element in TAF1 that can contribute to the stabilization of TFIID at RNAPII promoters during PIC formation. There are several possible explanations as to why TAF1 has evolved multiple DNA binding domains (DBDs). Firstly, several points of contact could aid in forming a secure connection between TFIID and a corresponding promoter long enough to recruit the other essential components of the transcriptional machinery. Additionally, multiple DBDs could confer TFIID the necessary flexibility to associate with a variety of core promoter sequences throughout the human genome, such that recognition of different promoters might involve different DNA binding domains. Last but not least, multiple DBDs might be important for the dynamic rearrangement of TFIID, which transitions from the conical form to the promoter bound form, as demonstrated by Cryo-EM studies^21,22^. Different DNA binding modules may be necessary to support this movement, properly orient TFIID, and load GTFs onto the core promoter. With these possibilities in mind, we raise two models for how TAF1 contributes to TFIID promoter recognition (Fig. 6). Either simultaneous binding where the WH and ZnK equally contribute to DNA binding or sequential binding where one DNA binding domain engages during the initial stages followed by a second DNA binding event in the rearranged conformation. Future studies using advanced high-resolution techniques will be needed to reveal the details of the elusive aspects of PIC assembly, as the current views may underestimate the complexity of TFIID promoter recognition.

Cyclin proteins are master regulators of the cell cycle. Their phasic expression mediates enzymatic activities required for growth and proliferation^45^. The impact of the TAF1 ZnK mutations on cyclin transcription is not due to an inability to incorporate into TFIID, implying this domain is not involved in TAF interactions or the core structure of TAF1, both important for TFIID assembly. While, this study focuses on the ability of ZnK to bind nucleic acids, our findings do not exclude the possibility the ZnK may interact with other regulatory proteins. The close proximity of the ZnK to the double bromodomain suggests the ZnK could play a role in facilitating the recognition of epigenetic markings, which could influence promoter association. There is growing evidence that zinc fingers can play a dual role: supporting DNA binding and facilitating protein interactions with histones or transcriptional activators, such as those found in CBP and ZYMND11^46–49^. However, our experimental approach cannot rule out this possibility considering our transcription assays were conducted with luciferase reporter plasmids not known to incorporate histones; despite this caveat, we conclude that the impact on cyclin transcription results from a defect in active PIC formation, attributed to loss of promoter binding by the ZnK mutant containing TFIID complex. The inability to stimulate transcription from the cyclin promoters would explain why the ZnK mutant cannot rescue the ts13 growth defect in the cell complementation assay.

The mechanisms proposed in this work may also provide some insight into the pathologies of human disease states associated with TAF1 mutations by GWAS. The cBioPortal and catalog of somatic mutations in cancer (COSMIC) databases denote several mutations in the conserved CCHC residues of TAF1 including: C1285R, C1288R, H1293N, C1300W/R. While it is unknown whether these mutations are causative, they reinforce the notion that these residues are crucial for proper TAF1 function in cells. TAF1 is essential for cell viability with deletion of the gene lethal to cells. For this reason, it is doubtful that disease-associated mutations completely obliterate TAF1 function and cause global transcriptional dysfunction. More likely, TAF1 function is altered in some subtle manner that results in aberrant transcription from select genes. The role of TFIID in proliferation and cell survival makes it of particular interest to cancer biology^50,51^.

In summary, we uncovered a second critical DNA binding domain in TAF1, the largest subunit of the TFIID general transcription factor. The conserved zinc knuckle is essential for the full *in vivo* function of TAF1. Mutations within this region do not disrupt the stability of TAF1 or its incorporation into TFIID, but instead interfere with its ability to associate with core promoters. The loss of promoter association would result in inefficient transcription initiation. We speculate that two motifs in TAF1 provide greater flexibility within TFIID for promoter DNA sequence recognition.

## Materials and Methods

### ts13 Complementation Assay

Mammalian TAF1 expression plasmids contain N-terminal HA-tagged human TAF1 coding sequence inserted downstream of the CMV promoter in CS2 + vector^52^. Point mutations in TAF1 were introduced by site-directed mutagenesis and confirmed by DNA sequencing (Supplemental Table S1,20). *ts*13 cells were grown at 33.5 °C in Dulbecco’s modified Eagles medium (Gibco) supplemented with 10% fetal bovine serum, 2 mM L-glutamine, and penicillin/streptomycin. For complementation assays, cells were seeded into 6-well plates, grown overnight to 70–80% confluency and transfected with 2 μg of CS2 + or TAF1 expression plasmids using polyethylenimine (PEI) transfection reagent (ratio 2.5:1 of PEI:DNA) according to previously described protocol^53^. Transfected cells were maintained for an additional 18–24 h at 33.5 °C, then either harvested for Western Blot analysis or shifted to nonpermissive temperature of 39.5 °C. Number of DAPI positive viable cells was determined after 36–48 h at 39.5 °C, and the percentage relative to WT-TAF1 transfected cells (given the value of 100%) was calculated.

### Luciferase Reporter Assay

Human cyclin D1 luciferase construct was constructed by subcloning the EcoRI to PvuII fragment of pD1-G065 (kindly provided by Yue Xiong) into the SmaI site of pGL2-basic as previously described^54^. The human cyclin A2 luciferase construct has been described^55^. The IMD luciferase construct is previously described in Juven-Gereshon *et al*.^56^ and a gift from J. Kadonaga. HEK293 cells were grown at 37 °C in Dulbecco’s modified Eagles medium (Gibco) supplemented with 10% fetal bovine serum, 2 mM L-glutamine, and penicillin/streptomycin. For luciferase assays, cells were seeded into 24-well plates, grown overnight to 60%-70% confluency and cotransfected with 100 ng of CS2 + or TAF1 expression constructs and 50 ng luciferase reporter construct using FuGene transfection reagent (ratio 2.5:1 of FuGene:DNA) according to the manufacturer’s protocol (Roche). Transfected cells were maintained an additional 36 h at 37 °C, after which cells were harvested and lysed in 100 μL of 1× Passive lysis buffer (Promega). Luciferase activity was measured according to manufacturer’s protocol (Promega) on Lumat LB 9507 (Berthold Technologies) and normalized for total protein. Transcriptional activity was expressed relative to CS2+/reporter construct transfected cells (set to 1).

### Cyclin D1 RT-qPCR

ts13 cells seeded into 35-mm dishes were grown at 33.5 °C to 60–70% confluency and transfected with 500 ng of CS2 + or HA-TAF1 expression plasmids using PEI (2.5:1 PEI:DNA ratio). Total RNA was isolated approximately 24–36 hours post-transfection and 1 μg RNA reverse transcribed in cDNA using iScript cDNA synthesis kit (BioRad). Cyclin D1 transcript levels were measuring by qPCR using SsoFast EvaGreen Supermix (BioRad) on the Applied Biosystems 7500Fast Real-Time PCR system. Relative transcript levels were determined by calculating 2^−ΔCt^.

### Chromatin Immunoprecipitation

HEK 293 cells were seeded into 10-cm dishes and transfected with CS2 + or TAF1 expression constructs and incubated for 24–36 h at 37 °C. Before harvesting, cells were cross-linked with 400 μl of 37% formaldehyde and analyzed as previously described^57^. In brief, cells were lysed in 0.5 mL immunoprecipitation (IP) buffer (50 mM Tris-HCl, pH 7.5, 150 mM NaCl, 5 mM EDTA, 1% Triton X-100, and 0.5% Nonidet P-40) containing protease inhibitors and sonicated 8 times for 20 s at 40% amplitude (Branson Digital Sonifier). Nuclear extract was incubated overnight at 4 °C with either anti-HA (clone 3F10, Sigma Aldrich) or mouse IgG (Abcam). Immunoprecipitated complexes were washed and eluted. Cross-links were reversed and DNA was isolated. Input DNA was purified from 100 μl extract. Fifty nanograms of input DNA and 2 μL of purified TAF1 bound DNA was amplified with SsoFast EvaGreen Supermix (Bio-Rad) using primers spanning the promoters of cyclin D1 and cyclin A2 (Supplemental Table S1). Quantitative PCR was performed on the Applied Biosystems 7500Fast Real-Time PCR system and the data expressed as percent input.

### TFIID and TAF1 Immunoprecipitation

For preparation of nuclear extracts, HEK293 cells were resuspended in buffer A (10 mM HEPES, pH 7.9, 1.5 mM MgCl_2_, 10 mM KCl, 0.5 mM DTT) and incubated on ice for 15 min. Cells were lysed by pushing through a 25-gauge needle 5 times. The crude nuclear pellet was isolated by centrifugation for 20 s at 12,000 g, resuspended in buffer C (20 mM HEPES, pH 7.9, 25% glycerol, 420 mM NaCl, 1.5 mM MgCl_2_, 0.2 mM EDTA, 0.5 mM phenylmethylsulfonyl fluoride, 0.5 mM DTT), and incubated for 30 min at 4 °C. After centrifugation for 5 min at 12,000 g, the supernatant/nuclear extract was incubated with anti-TAF4 (mAb 3A6, ref.^58^) or anti-HA antibody overnight at 4 °C. Ten microliters of protein A–Sepharose CL-4B (GE Healthcare) was added, and the samples were nutated for 2 h at 4 °C. Isolated proteins were washed 5 times with Buffer C and analyzed by silver stain or immunoblotting using antibodies against HA (clone 3F10, Sigma Aldrich), TAF1 bromodomain (Ab1230, ref.^59^), TBP (gift from R. Tjian, ref.^5^), TAF4 (mAb 3A6), and TAF5 (mAb 6C1, unpublished data).

### Protein Purification

For expression of His-tagged TAF1 proteins, cDNA of TAF1 (ZnA-1234–1371, ZnC-1234–1303, ZnD-1256–1303) was PCR amplified from CS2 + containing full length TAF1 and cloned into pAL-GB1 using ligase independent cloning; site-directed mutagenesis was used to introduce point mutations into ZnC and ZnD constructs (Supplemental Table S1). TAF1 ZnK expression constructs were transformed into BL21* cells for protein expression. Starter cultures of 10 mL were diluted into 1 L LB containing 30 μg/ml chloramphenicol, and cells were grown to an optical density of 0.8–1.0 at 600 nm and chilled on ice for 1 h. TAF1 expression was induced by addition of 0.2 mM IPTG (isopropyl–D-thiogalactopyranoside) for 18 h at 16 °C. Cells were harvested by centrifugation and lysed by pulse-sonication in 50 mM Tris-HCl, pH 8.0, 300 mM NaCl, 5 mM imidazole, 5% glycerol, 0.05% Triton-X, 1 mM DTT supplemented with protease inhibitors. His-GB1-TAF1 was purified using Ni-nitrilotriacetic acid (NTA)- agarose (Qiagen), washed with Buffer A (50 mM Tris-HCl, pH 8.0, 150 mM NaCl, 10 mM imidazole, 5% glycerol, 1 mM DTT) followed by a high salt wash (50 mM Tris-HCl, pH 8.0, 1 M NaCl, 5% glycerol, 1 mM DTT) and a final wash with Buffer A. Protein was eluted with 50 mM Tris-HCl, pH 8.0, 150 mM NaCl, 200 mM imidazole, 5% glycerol, 1 mM DTT. For proteolytic digestion and electrophoretic mobility shift assays, ZnA was further purified by cation exchange (GE Healthcare) after off-column cleavage by the tobacco etch virus (TEV) protease. Proteins for biolayer interferometry were further purified by size-exclusion chromatography (GE Healthcare) using 10 mM HEPES, pH 7.9, 150 mM NaCl, 1 mM DTT.

### Electrophoretic Mobility Shift Assay

Purified TAF1 (ZnA-1234–1371) (7.6–61.3 pmoles) purified from *E. coli* was incubated with 4 ng of ^32^P 5′-end labeled DNA in 10 mM HEPES pH 7.9, 5 mM MgCl_2_, 100 mM NaCl, 10% glycerol, 20 mM tetrasodium pyrophosphate, 0.2 mM dI:dC for 1 h at 25 °C. For gel electrophoresis, 6× loading buffer (20% Ficoll, 0.025% bromophenol blue) was added and binding reactions were loaded onto nondenaturing 5% polyacrylamide (37.5:1 acrylamide:bis) pre-run in 0.5× TBE at 100 V for 30 min. Samples were resolved for 1.5 h at 100 V and shifted complexes detected by autoradiography. The sequences of DNAs used for EMSA are provided in Supplemental Table S1.

### Biolayer Interferometry

5′-biotinylated oligonucleotides were annealed to their complement (IDT, USA) in 1× binding buffer (10 mM HEPES pH 7.9, 100 mM NaCl, 10 mM MgCl_2_, 1 mM DTT) by heating at 72 °C for 5 minutes and slow cooling to 25 °C. Double-stranded oligos (Supplemental Table S1) were purified by extraction from agarose after gel electrophoresis then ethanol precipitated. Purified biotinylated double-stranded oligos were loaded onto streptavidin-coated (SA) sensors and the interactions between the DNA coated probes and TAF1 proteins were measured using ForteBio Octet RED96 system (Pall Life Sciences). For loading, SA sensors were prewetted in assay buffer (10 mM HEPES pH 7.9, 5 mM MgCl_2_, 100 mM NaCl, 10% glycerol, 0.1% ovalbumin, 0.2 mM dI:dC) for 30 min then dipped into individual wells on 96-well plates (200 μL/well) containing biotinylated DNA (100 nM) in assay buffer. Unbound DNA was removed by washing and exposed streptavidin blocked with biocytin. Protein was diluted in assay buffer to 3 μM, 1 μM, 333 nm 111 nm, 37 nm, and 12 nm. Association of the protein to the probe was observed for 300 s followed by dissociation in assay buffer for 300 s. Throughout loading, association and dissociation sensors were shaking (1,000 rpm) at a constant 30 °C to promote specific interaction and to reduce non-specific binding. A reference sensor (no biotinylated DNA loaded) was included and subjected to the same procedure to control for non-specific interactions. Sensors were regenerated (10 mM HEPES pH 7.9, 1 M NaCl) between experiments and a new baseline established. Reference-subtracted data was used to calculate the equilibrium dissociation constant (K_d_) by steady-state response analysis assuming 1:1 kinetics.

### DNA Protection Assay

TAF1 (aa 1234–1371) was exposed to limited proteolytic digestion in the presence and absence of DNA (IMD) to determine DNA-bound stabilized regions. TAF1 (625 pmol) was incubated with or without DNA (700 pmol) in 10 mM HEPES pH 7.9, 5 mM MgCl_2_, 100 mM NaCl, 10% glycerol, 20 mM tetrasodium pyrophosphate, 0.2 mM dI:dC) for 1 h at 25 °C. Subtilisin (0.002, 0.006, 0.02, 0.06, 0.2, and 0.6 μg/ml) was added to each sample and incubated overnight on ice at 4 °C. Samples were divided in two and analyzed as follows: SDS-PAGE, coomassie stained, and scanned for analysis; SDS-PAGE, transferred to PVDF membrane, coomassie stained and stabilized fragments excised and N-terminally sequenced.

### Data availability

All data generated or analyzed during this study are included in this published article (and its Supplementary Information files).

## Electronic supplementary material

Supplemental Figures and Table
