# Supplementary material for: Zinc knuckle of TAF1 is a DNA binding module critical for TFIID promoter occupancy
Source: Sci Rep. 2018 Mar 15;8:4630. doi: 10.1038/s41598-018-22879-5 (PMC5854669; doi:10.1038/s41598-018-22879-5)
Supplement: Supplementary file 1 — Supplemental Figures and Table [file 41598_2018_22879_MOESM1_ESM.pdf]

Zinc knuckle of TAF1 is a DNA binding module critical for TFIID promoter occupancy.

Elizabeth C. Curran[1], Hui Wang[1,2], Thomas R. Hinds[1], Ning Zheng[1,2], & Edith H. Wang[1]

Supplementary Information

Supplemental Figure S1

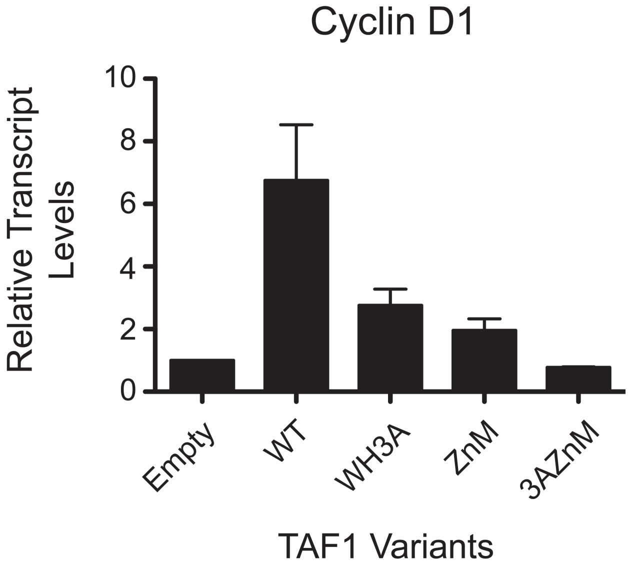

Supplemental Figure S1: Cyclin D1 gene expression in ts13 cells. Endogenous cyclin D1 transcript levels in ts13 cells were measured by RT-qPCR after expression of TAF1 variants at permissive temperature (33.5˚C) for 24-36 hours. n=2, Error bars represent standard deviation.

Supplemental Figure S2

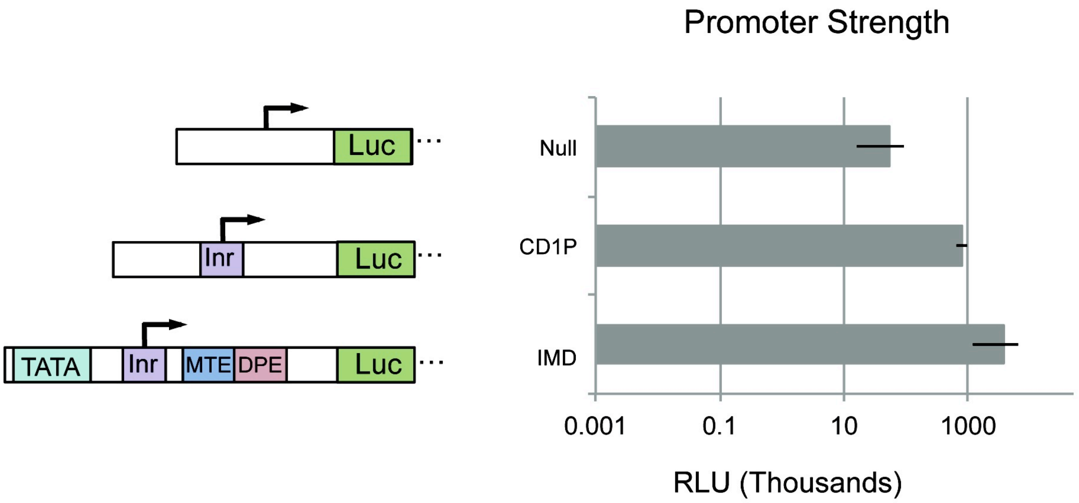

Supplemental Figure S2: Promoter strength assessment. Luciferase reporter assay of pGL2 Basic (Null), cyclin D1 (CDP1), and IMD promoter driven reporter constructs. Signal expressed on a log scale in relative light units (n=4).

Supplemental Figure S3

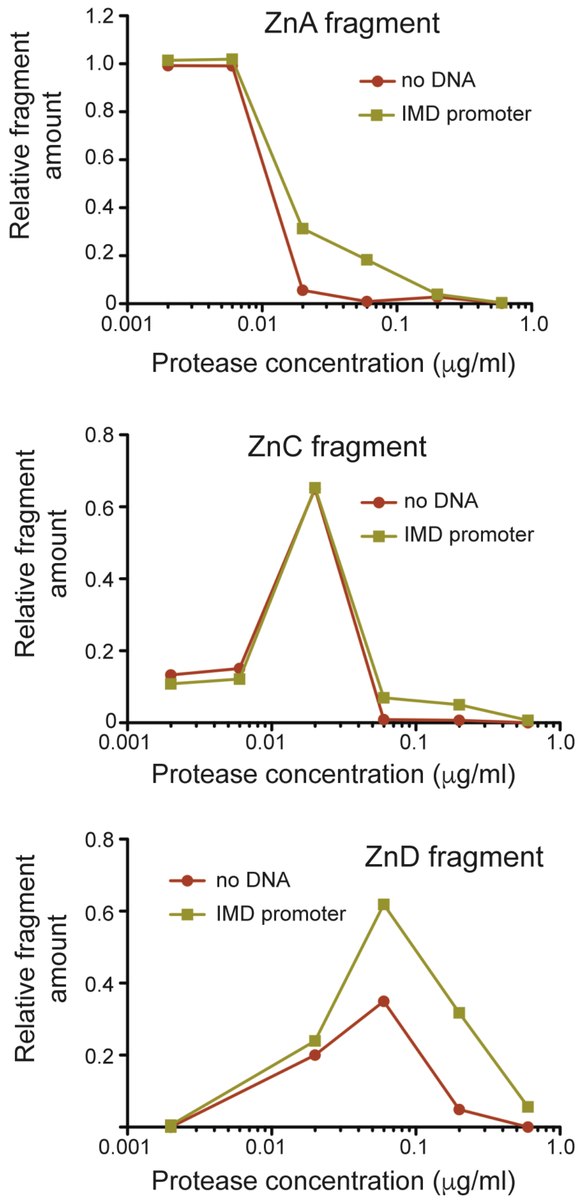

Supplemental Figure S3: Quantification of DNA protected ZnK fragments from protease digestion shown in Figure 5A. The intensity of different ZnK protein fragments in coomassie stained gels from Figure 5A was quantified using ImageJ software. The amount of each fragment relative to ZnA band detected in the absence of protease digestion was determined and plotted against protease concentration. Inclusion of IMD promoter lead to increased fragment levels indicative of protection from proteolysis.

Supplemental Figure S4

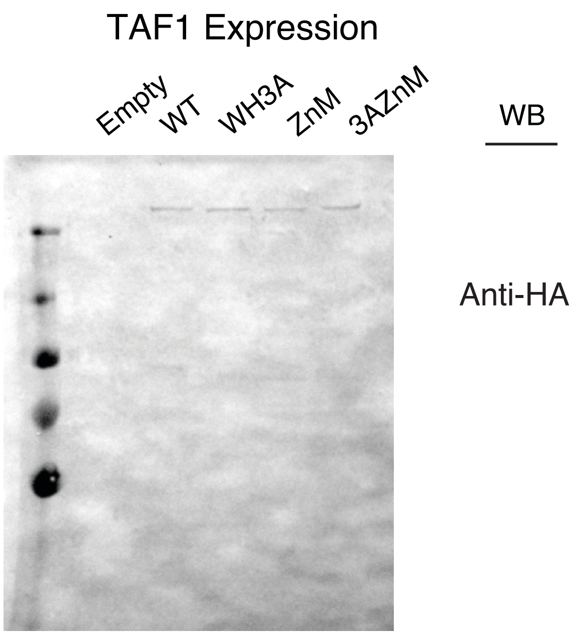

Supplemental Figure S4: Full-length Western Blot used to generate Figure 2C. Exogenously expressed HA-TAF1 was enriched using anti-TAF1 double bromodomain antibody (Ab1230, Ref. 53) and detected using anti-HA antibody (clone 3F10, Sigma Aldrich).

Supplemental Figure S5

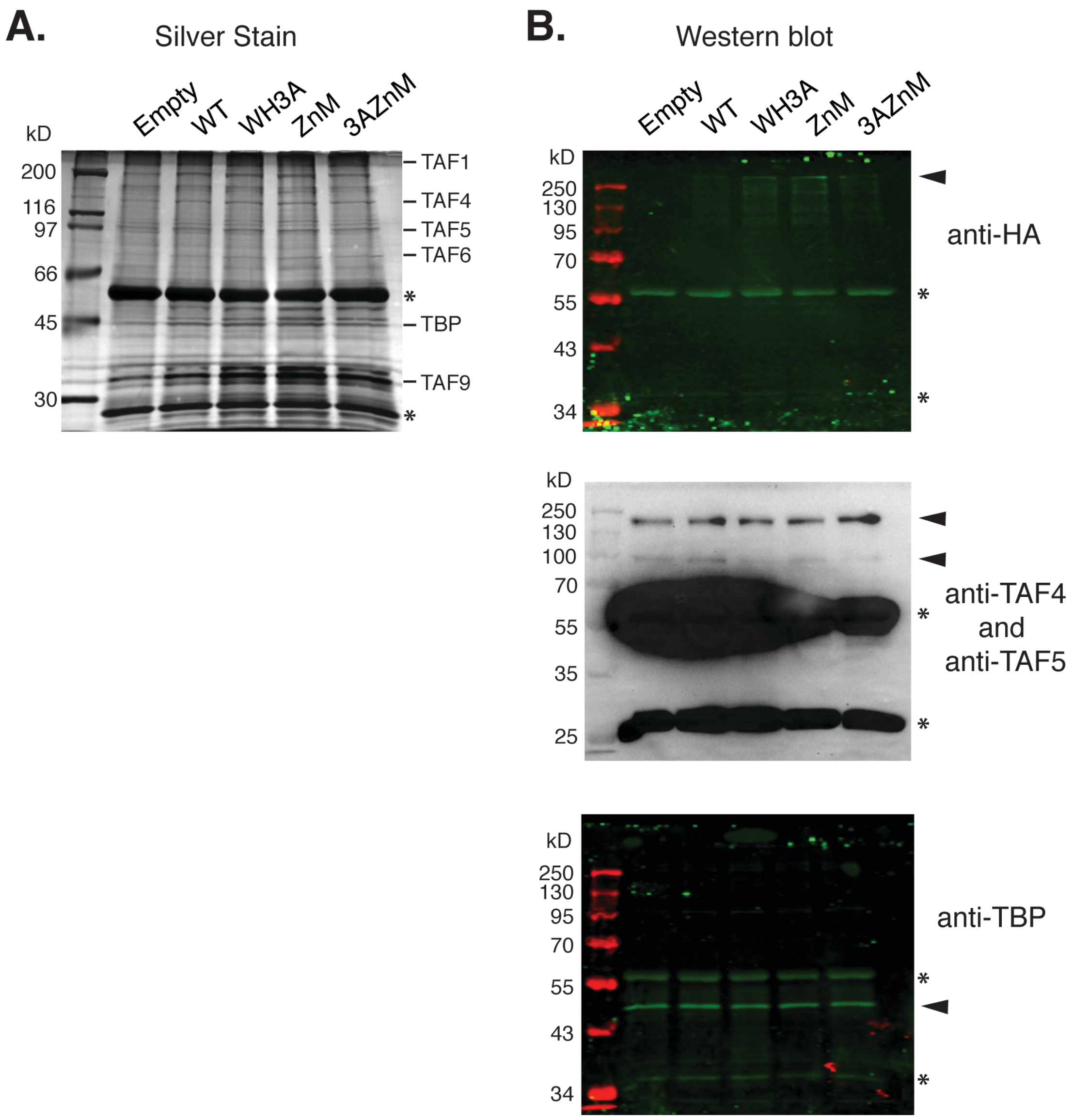

Supplemental Figure S5: Full-length Western Blots used to generate Figure 3A. TFIID was isolated using anti-TAF4 antibody (mAb 3A6, Ref. 52). After separation on 10% SDS-PAG, immunoprecipitated proteins were visualized by silver stain (A) or Western blot (B). For Western blotting, HA-TAF1 was detected using anti-HA rat antibody (clone 3F10, Sigma Aldrich) followed by TBP using an anti-TBP rabbit polyclonal antibody (5). Both HA-TAF1 and TBP were imaged on Li-Cor Odyssey infrared imaging system. Blot was re-probed with anti-TAF4 and anti-TAF5 mouse monoclonal antibodies and imaged by chemiluminescence on ChemiDoc XRS+ System. Bands representing IgG heavy and light chains are indicated by the asterisks. The position of TFIID subunits are labeled or indicated by the arrowheads. Molecular weight standards are labeled on the left side.

Supplemental Table S1

Oligonucleotides		
**Name**	**Use**	**Nucleotide Sequence (5'-3' only sense strand listed)**
Cysmut	Mutagenesis	5'-ctgccctaaaactgaaagctggggcagctggtgccattggaca c-3'
RKmut	Mutagenesis	5'-tgccattggacacatggcgactaacgcattctgccccctctattat-3'
3AWH	Mutagenesis	5'-gacgacaggctagcgctctgcgctgac-3'
Cyclin D1 -91 For	qPCR	5'-cgtcacacggactacagggg-3'
Cyclin D1 +29 Rev	qPCR	5'-cgctcggctctcgcttctgc-3'
Cyclin A2 -143 For	qPCR	5'-tcc agcgggctgctcgctgc-3'
Cyclin A2 -41 Rev	qPCR	5'- ctcgagaccacgcagggccgagga-3'
SGH-Cyclin D1 RT-For	RT-qPCR	5'-ccctccgtgtcttacttcaag-3'
Cyclin D1 RT-Rev	RT-qPCR	5'-aggaagcggtccaggtagtt-3'
IMD fragment of Super Core Promoter (IMD)	DNA Binding	5'-gtcctcagtcgcgatcgaacactcgagccgagcagacgtgccta-3'
Cyclin D1 core promoter (CD1)	DNA Binding	5'-aggggagttttgttgaagttgcaaagtcctggagcctccagagggctgtcg- 3'
Random DNA	DNA Binding	5'-gaa tgtcgcgaaggcagcgttcgcgaagacagcaaagaag-3'

Supplemental Table S1: Sequence of mutagenesis primers, quantitative PCR primers used in ChIP analysis, primers for RT-qPCR analysis in hamster cells, and DNA binding probes.

Supplemental Table S2

	Protein Intensity				HA-TAF1 relative to:		
TAF1 variant	*HA-TAF1*	*TAF4*	*TBP*	*IgG_H*	*TAF4*	*TBP*	*IgG_H*
Empty	0.36	12815	146.47	17.69	1.0	1.0	1.0
WT	3.58	15531	161.08	19.62	8.21	9.04	8.97
WH3A	3.93	13329	112.45	16.84	10.50	14.22	11.47
ZnM	8.46	12688	115.85	14.59	23.74	29.71	28.49
3AZnM	4.46	23699	133.85	17.76	6.7	13.56	12.34

Supplemental Table S2: Quantitation of immunoprecipitated TFIID subunits. The relative intensity of TFIID subunits and anti-TAF4 heavy chain (IgGH) in western blots from Figure 3A was quantified using Li-COR Odessey (HA-TAF1, TBP, IgG_H) or ImageJ (TAF4) software. The amount of each HA-TAF1 variant relative to different TFIID subunits and IgG_H was determined and normalized to Empty vector, given a value of 1.0.
